# Linagliptin and telmisartan induced effects on renal and urinary exosomal miRNA expression in rats with 5/6 nephrectomy

**DOI:** 10.1038/s41598-020-60336-4

**Published:** 2020-02-25

**Authors:** Denis Delić, Franziska Wiech, Richard Urquhart, Ogsen Gabrielyan, Kathrin Rieber, Marcel Rolser, Oleg Tsuprykov, Ahmed A. Hasan, Bernhard K. Krämer, Patrick Baum, Andreas Köhler, Florian Gantner, Michael Mark, Berthold Hocher, Thomas Klein

**Affiliations:** 1 Boehringer Ingelheim Pharma GmbH & Co. KG, Translational Medicine & Clinical Pharmacology, Biberach, Germany; 2 Boehringer Ingelheim Pharma GmbH & Co. KG, Cardiometabolic Diseases Research, Biberach, Germany; 3 Fifth Department of Medicine (Nephrology/Endocrinology/Rheumatology), University Medical Centre Mannheim, University of Heidelberg, Heidelberg, Germany; 4 Department of Biochemistry, Faculty of Pharmacy, Zagazig University, Zagazig, Egypt

**Keywords:** Biochemistry, Molecular biology

## Abstract

Dipeptidyl peptidase 4 inhibitors and angiotensin II receptor blockers attenuate chronic kidney disease progression in experimental diabetic and non-diabetic nephropathy in a blood pressure and glucose independent manner, but the exact molecular mechanisms remain unclear. MicroRNAs (miRNAs) are short, non-coding RNA species that are important post-transcriptional regulators of gene expression and play an important role in the pathogenesis of nephropathy. miRNAs are present in urine in a remarkably stable form, packaged in extracellular vesicles. Here, we investigated linagliptin and telmisartan induced effects on renal and urinary exosomal miRNA expression in 5/6 nephrectomized rats. In the present study, renal miRNA profiling was conducted using the Nanostring nCounter technology and mRNA profiling using RNA sequencing from the following groups of rats: sham operated plus placebo; 5/6 nephrectomy plus placebo; 5/6 nephrectomy plus telmisartan; and 5/6 nephrectomy plus linagliptin. TaqMan Array miRNA Cards were used to evaluate which of the deregulated miRNAs in the kidney are present in urinary exosomes. In kidneys from 5/6 nephrectomized rats, the expression of 13 miRNAs was significantly increased (>1.5-fold, *P* < 0.05), whereas the expression of 7 miRNAs was significantly decreased (>1.5-fold, *P* < 0.05). Most of the deregulated miRNA species are implicated in endothelial-to-mesenchymal transition and inflammatory processes. Both telmisartan and linagliptin suppressed the induction of pro-fibrotic miRNAs, such as miR-199a-3p, and restored levels of anti-fibrotic miR-29c. In conclusion, the linagliptin and telmisartan-induced restorative effects on miR-29c expression were reflected in urinary exosomes, suggesting that miRNA profiling of urinary exosomes might be used as a biomarker for CKD progression and monitoring of treatment effects.

## Introduction

Chronic kidney disease (CKD) is a major global health problem associated with significant morbidity and mortality^1^. Hypertension and diabetes mellitus are known to be the main contributors to the development and progression of CKD in developed countries^2^. Reducing blood pressure using angiotensin II receptor blockers or angiotensin-converting enzyme is the first-line therapy for delaying CKD progression^3^. Despite the control of the renin-angiotensin system glycemic control is essential for therapies combating diabetic nephropathy.

Dipeptidyl peptidase (DPP)-4 inhibitors are indicated for the treatment of diabetes, however beneficial effects independent of glucose and renal blood pressure have been shown in animal models of both diabetic^4^ and non-diabetic CKD^5^. It is well known that the antidiabetic effects of DPP-4 inhibitors are mediated via increases in levels of the incretin hormones glucagon-like peptide 1 (GLP-1) and glucose-dependent insulinotropic polypeptide (GIP)^6^. Moreover, recent experimental data demonstrated that linagliptin exerts anti-inflammatory^7^, anti-oxidant^8^ and anti-fibrotic^9^ effects, however the exact underlying renoprotective molecular mechanisms remain unclear.

In diabetic and non-diabetic CKD pathogenesis TGF-β signaling is known to induce the synthesis and accumulation of extracellular matrix (ECM) leading to fibrosis and hypertrophy in various kidney cell types^10^. TGF-β-induced expression of ECM genes during the development of diabetic nephropathy is mediated by several miRNA species. The functional crosslink between TGF-β and responsive miRNA expression has been demonstrated in renal cells, such as proximal tubular epithelial cells, mesangial cells and podocytes. Increased glomerular miR-21 level are positively associated with the albumin-creatinine ratio (ACR) in patients with diabetic nephropathy and it was postulated that altered miRNA-21 level might serve as an indicator of podocyte damage^11^. Another prominent pro-fibrotic miRNA is miR-199a which is involved in the regulation of TGF-β signaling^12^. Increased renal expression of miR-199a was observed in the unilateral ureteral obstruction mouse model and inhibits the expression of caveolin 1 which is a critical process in the activation of fibroblasts by TGF-β^13^. In contrast, expression of kidney damage protective miRNAs, such as members of the miR-200-, miR-30- and miR-29-families, is decreased in various kidney cell types^10^. miR-29 family members are down-regulated in CKD such as diabetic nephropathy, focal glomerulosclerosis, membraneous nephropathy and IgA nephropathy^14,15^. *In vitro* studies revealed that inhibition of overexpression of and knockdown of miR-29 enhanced TGF-β induced expression of collagens type I and III by renal tubular cells^16^. Streptozotocin-induced diabetic miR-29 transgenic mice showed improved renal function and better podocyte viability whereas knockdown of miR-29 promoted podocyte apoptosis, proteinuria and subsequent renal dysfunction^17^. The DPP-4 inhibitor linagliptin restored the level of renal miR-29s, which was associated with the inhibition of TGF-β-induced endothelial to mesenchymal transition (EndMT) in the kidneys of diabetic mice^9^.

Extracellular vesicles, such as exosomes, are secreted in large quantities from all nephron segments^18^ into the urine and may provide valuable insights into renal pathophysiology. Urinary exosomal miRNA content is altered in patients with focal segmental glomerulosclerosis^19^, in type I diabetic patients with incipient diabetic nephropathy^20^, and in type II diabetic patients with diabetic nephropathy^21^. Moreover, it was shown that altered urinary exosomal miR-29c level might potentially serve as a predictor of early fibrosis in lupus nephritis^22^.

In the present study we investigated the effects of linagliptin and compared with those of telmisartan which is one of the most commonly used ARBs on dysregulated miRNAs in the kidney in the rat 5/6 nephrectomy model, one of the most well-established experimental non-diabetic CKD models. We aimed to identify those effects on urinary exosomal miRNA level which might serve as potential novel biomarkers for monitoring both disease progression and treatment effects.

## Materials and Methods

### Experimental design

The aim of this study was to identify the effects of telmisartan and linagliptin on miRNAs differentially expressed in kidneys from 5/6 Nx rats and to assess whether these effects can be accurately quantified in urinary exosomes. As a first, step large-scale miRNA and mRNA expression profiles from the same renal tissue samples were evaluated using the Nanostring and RNA sequencing platforms, respectively. Changes in miRNA expression level were identified by a moderated t-test, evaluating only those miRNAs with at least 1.5-fold expression changes at a *P*-value <0.05. In addition, *P*-values were further corrected for multiple testing according to Benjamini-Hochberg. Functional annotations of these miRNAs were searched in several databases including miRWalk (http://www.umm.uni-heidelberg.de/apps/zmf/mirwalk), TargetScan (http://www.targetscan.org), miRBase (http://www.mirbase.org), and PubMed (http://www.ncbi.nlm.nih.gov/pubmed). Inverse correlated miRNA-target mRNA pairs were identified using Ingenuity Pathway Analysis (http://www.qiagenbioinformatics.com). Highly predicted target mRNAs with a Spearman´s correlation coefficient of at least −0.7 were further assessed. Quantitative real-time polymerase chain reaction (PCR) was then used to detect differentially expressed renal miRNAs in urinary exosomes.

### Animals

Eight week old Wistar rats were purchased from Charles River Laboratories International, Inc. (Wilmington, MA). This study (animal studies and protocols) was approved by the Committee on the Ethics of Animal Experiments (Landesamt fuer Gesundheit und Soziales), Berlin, Germany and is in accordance with University Guidelines for Use of Laboratory Animals. The animals were assigned into 4 groups: sham operation (sham) + placebo; 5/6 nephrectomy (5/6 Nx) + placebo; 5/6 Nx + telmisartan; 5/6 Nx + linagliptin. The Nx operation was performed as follows: uninephrectomy at Week 1, followed at Week 3 by amputation of the poles of the remaining kidney. Sham operations were performed at the same time points. Linagliptin (83 mg/kg/day in chow) and telmisartan (5 mg/kg/day in drinking water) were administered from Day 4 after the second surgery for 13 weeks. Immediately after this treatment period animals were sacrificed and organs were harvested. Urine albumin-to-creatine ratio (UACR), interstitial fibrosis, plasma DPP-4 activity and urinary DPP-4 activity/creatinine ratios of the experimental groups are summarized in Table S1. Further physiology and biochemistry parameters of animals were described in detail by Tsuprykov *et al*.^23^ and isolated total RNA was used for the following experiments.

### Nanostring analysis

Total RNA from kidney tissue was extracted using RNeasy Fibrous Tissue Mini Kit (Qiagen, Hilden, Germany). RNA purity and quantity were assessed by NanoDrop 2000 (ThermoScientific, Waltham, MA, USA). Total RNA (100 ng) was used to assess the miRNA expression using the nCounter Rat v1.5 miRNA CodeSet (based on miRBase v17, Nanostring Technologies, Seattle, WA, USA) which contains a library of 420 probes. The purified complexes were quantified on the nCounter Digital Analyzer and analyzed by nSolver software (v1.1; Nanostring Technologies, Seattle, WA). The exact data analysis procedure can be found at: http://www.NanoString.com/media/pdf/MAN_nCounter_Gene_Expression_Data_Analysis_Guidelines.pdf.

### mRNA library preparation, sequencing and data analysis

RNA sequencing libraries were prepared using Illumina’s TruSeq RNA Sample Prep Kit - v2 (Illumina Inc.) according to the manufacturer’s instructions. The library concentrations were then quantified with the Quant-iT PicoGreen dsDNA Assay Kit (Quant-iT) using CLARIOstar (BMG LABTECH) and the library quality was determined by checking cDNA fragment size using a DNA1000 Kit on the Agilent Bioanalyzer 2100 (Agilent Tech Inc.). Libraries were then normalized to 2 nM and subjected to cluster generation on a cBot system followed by single-read sequencing of 52 bp on an Illumina HiSeq. 2000 instrument (Illumina Inc.). Quality check (QC) of the obtained reads was performed using FASTQC v0.10.1. Based on RPKMs/FPKMs as obtained in the subsequent analysis (1.6.2), principal component analysis (PCA) and hierarchical clustering analysis were further used to identify outliers. Outliers were removed from subsequent analysis. Reads were then mapped to the human reference genome hg19 (GRCh37 Ensembl v. 70, primary assembly) using STAR v2.3.0e. Mapped reads were QCed using RNA-SeQC v1.1.7. Gene expression intensities are represented as either uRPKM/uFPKM or mRPKM/mFPKM values and these were calculated based on Ensembl v70 gene annotations using cufflinks v2.2.1 for Linux_x86_64. RPKM/FPKM values account for the different lengths of genes as well as different number of reads measured for the respective sample. Only those reads that could unambiguously be assigned to the respective gene were considered in the analysis to determine uRPKM/uFPKM values. For mRPKM/mFPKM values multiple mapping reads were also considered. SAM to BAM conversion was done using picard-tools-1.77. Differential expression analysis was conducted based on read counts per gene. Read counts per gene (Ensembl v70 gene annotations) were either obtained using htseq-count from HTSeq v0.5.3p9 and samtools v0.1.18 or based on cufflinks v2.2.1 results. Fold changes and their respective significance were computed based on the read counts obtained for each gene using R and Bioconductor packages edgeR, DESeq2 or voom in conjunction with limma.

### Integrated analysis of miRNA and mRNA expression

Spearman correlation coefficients (r_s_) between mRNA and miRNA expression values for each sample were calculated. Correlation coefficients ranged from −1 to 1 and r_s_ < −0.7 were considered for further evaluation.

### Urinary exosomal miRNA expression

The animals were placed in metabolic cages to obtain 24-hour urine samples. Urine samples from three animals were pooled due to the limited amount of urine, resulting in 4 samples in total per experimental group. Urinary exosomal miRNAs were isolated using the exoRNeasy Serum/Plasma Maxi Kit (QIAGEN, Hilden, Germany) and the expression of the miRNAs was screened using Taqman Fast Advanced MasterMix (Applied Biosystems) and the rodent Taqman miRNA Array, Card A V.2.0 (Applied Biosystems). The gene expression analysis was run on a SDS7900HT real-time PCR system (Applied Biosystems by ThermoFisher Scientific). TaqMan MicroRNA Reverse Transcription Kit (Applied Biosystems) and Megaplex RT Primers, Rodent Pool A v2.1 (Applied Biosystems) were used to reverse transcribe miRNAs. The RT products were pre-amplified using Megaplex RT Pre-Amp Primers, Rodent Pool A v2.1 (Applied Biosystems). U6snRNA was used for normalization. PCR reactions were performed with the TaqMan gene expression master mix (Life Technologies) according to the manufacturer´s protocol on a SDS7900HT real-time PCR System. Raw Ct values were calculated using the SDS software v2.4. Threshold cycle (Ct) values > 35 were excluded from any calculations. Fold change of expression was calculated using the comparative Ct method (2^−ΔΔct^)^24^.

### Statistical analysis

Differential expression was calculated applying the linear models approach incorporated in the limma software package^25,26^ using Tibco Spotfire version 6.5.2 (TIBCO Software, Palo Alto, USA). miRNAs >1.5-fold and mRNAs >1.5-fold differentially expressed between the compared groups with a *P*-value adjusted for multiple testing <0.05 were considered to be significantly differentially expressed. Adjustment of *P*-values for multiple testing was performed according to Benjamini and Hochberg^27^. Heatmaps, principal component analyses and volcano plots were generated via Tibco Spotfire (TIBCO Software). Hierarchical clustering was calculated using unweighted pair group method with arithmetic mean as the default clustering method and Euclidean distance measure.

Statistical analysis for the PCR data was performed using ANOVA and Tukey’s multiple comparisons test. For group-wise comparisons, expression was considered significantly changed if the respective fold change was >1.5 and *P*-value <0.05.

## Results

### Effects of telmisartan and linagliptin on differentially expressed miRNAs in kidneys from 5/6 Nx rats

**Figure 1:**
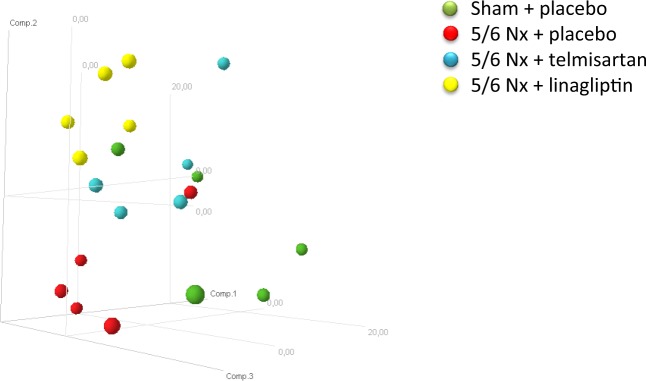
Principal component analysis of miRNA expression profiling results. Displayed are the first three major components from the principal component analysis. (green = sham+placebo; red = 5/6 Nx + placebo; blue = 5/6 Nx + telmisartan; yellow = 5/6 Nx + linagliptin).

**Figure 2:**
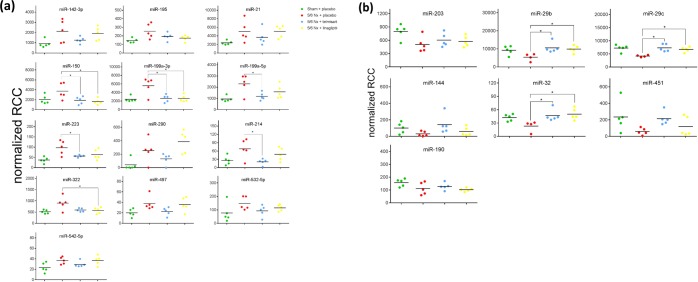
Up- (**a**) and down-regulated (**b**) renal miRNA expression. MiRNA levels of sham-operated (green), placebo-treated 5/6 Nx (red), telmisartan-treated (blue) and linagliptin-treated 5/6 Nx (yellow) rats revealed by Nanostring analysis. Absolute values are displayed for each animal by normalized reporter cell counts (RCC).

Principal component analysis revealed that the miRNA expression profiles were relatively similar within each experimental group whereas the respective groups clustered separately (Fig. 1). Compared with sham-operated animals, in 5/6 Nx-placebo-treated rats, the renal expression of the 13 miRNAs miR-142-3p, miR-195, miR-21, miR-150, miR-199a-3p, miR-199a-5p, miR-214, miR-223, miR-290, miR-322, miR-497, miR-532-5p and miR-542-5p was significantly up-regulated (Fig. 2a), whereas the renal expression of the 7 miRNAs miR-144, miR-190, miR-203, miR-29b, miR-29c, miR-32 and miR-451was significantly down-regulated (Fig. 2b). Telmisartan significantly ameliorated the expression of miR-150, miR-199a-5p, miR-199a-3p, miR-223 and miR-214, whereas linagliptin significantly ameliorated the expression of miR-150, miR-199a-3p and miR-322 compared to the 5/6 Nx placebo group (Fig. 2a). Significant restoring effects on the expression of miR-29b, miR-29c and miR-32 were observed for both telmisartan- and linagliptin-treated 5/6 Nx rats compared to placebo-treated 5/6 Nx rats. The degree of fold changes of distinct up- and down-regulated renal miRNA expression profiles correlates with the degree of renal interstitial fibrosis (Supplementary data, Figs. S1 and S2). The functions of the deregulated miRNAs based on published data are summarized in Table 1. Most of the deregulated miRNAs in the kidney from 5/6 Nx + placebo rats were involved in fibrotic processes, such as the 8 miRNAs miR-21, miR-29b, miR-29c, miR-150, miR-199a-3p, miR-199a-5p, miR-203 and miR-214. The 6 miRNAs miR-142-3p, miR-144, miR-223, miR-290, miR-322 and miR-451 are involved in cell development and/or differentiation while the remaining 6 miRNAs miR-190, miR-195, miR-32, miR-497, miR-532-5p and miR-542-5p are involved in cell cycle processes mostly via acting as tumor suppressors (Table 1).

**Table 1:** Effects of telmisartan and linagliptin on differentially expressed miRNAs in kidney from 5/6 Nx rats.

miRNA	Sham + placebo	5/6 Nx + placebo	5/6 Nx + telmisartan	5/6 Nx + linagliptin	Functions	PMID
**upregulated miRNAs**						
miR-142-3p	1 ± 0.18	2.17 ± 0.54^a^	1.42 ± 0.15	2.10 ± 0.32	Regulates formation and differentiation of hematopoietic stem cells.	24165894
miR-195	1 ± 0.07	1.76 ± 0.26^a^	1.32 ± 0.13	1.20 ± 0.10	MicroRNA-195 promotes apoptosis in mouse podocytes via enhanced caspase activity driven by BCL2 insufficiency.	22123611
miR-21	1 ± 0.08	2.11 ± 0.46^a^	1.54 ± 0.34	2.12 ± 0.26	Upregulated during fibrosis and represent prominent regulatory factors of TGFβ signaling. In kidney biopsies of diabetic patients, miR-21 correlated with tubulointerstitial injury. miR-21 antagonism *in vitro* and *in vivo* in streptozotocin-induced diabetic mice decreased mesangial expansion, interstitial fibrosis, macrophage infiltration, podocyte loss, albuminuria, and fibrotic- and inflammatory gene expression.	28129112
miR-150	1 ± 0.19	1.83 ± 0.35^a^	0.92 ± 0.16^b^	0.80 ± 0.13^c^	Promotes renal fibrosis in lupus nephritis by downregulating SOCS1.	23723424
miR-199a-3p	1 ± 0.12	2.20 ± 0.37^a^	1.06 ± 0.15^b^	1.07 ± 0.14^c^	p53 induces miR199a-3p to suppress SOCS7 for STAT3 activation and renal fibrosis.	28240316
miR-199a-5p	1 ± 0.11	2.30 ± 0.39^a^	1.24 ± 0.16^b^	1.62 ± 0.28	miR-199a-5p is upregulated during fibrogenic response to tissue injury.	23459460
miR-223	1 ± 0.17	2.62 ± 0.42^a^	1.51 ± 0.06^b^	1.72 ± 0.30	Acts as an important modulator of myeloid cell development and functions.	27704281
miR-290	1 ± 0.85	5.99 ± 1.85^a^	3.10 ± 0.83	9.16 ± 1.78	Highly expressed in embryonic stem cells.	25030899
miR-214	1 ± 0.28	2.62 ± 0.59^a^	0.81 ± 0.18^b^	1.85 ± 0.41	Promotes renal tubular epithelial cell mesenchymal transition and renal fibrosis.	29277613
miR-322	1 ± 0.07	1.74 ± 0.26^a^	1.15 ± 0.07	1.11 ± 0.11^c^	miR-322 targets key receptors of insulin signaling pathway and sirtuin 4. miR-322 promotes mitochondrial respiratory chain and fatty acid metabolism genes.	26775030
miR-497	1 ± 0.17	1.89 ± 0.30^a^	1.13 ± 0.17	1.81 ± 0.30	MicroRNA-497 suppresses renal cell carcinoma by targeting VEGFR-2 in ACHN cells.	28465356
miR-532-5p	1 ± 0.41	2.46 ± 0.28^a^	1.41 ± 0.24	1.96 ± 0.13	Acts as tumor suppressor in different cancer types.	26807173
miR-542-5p	1 ± 0.17	1.56 ± 0.13^a^	1.25 ± 0.11	1.57 ± 0.17	Acts as tumor suppressor in different cancer types.	28927388
**downregulated miRNAs**						
miR-203	1 ± 0.09	0.62 ± 0.09^a^	0.75 ± 0.08	0.72 ± 0.06	miR-203 downregulation has been reported in rat and human fibrotic liver tissue.	28586069
miR-29b	1 ± 0.12	0.63 ± 0.11^a^	1.15 ± 0.13^b^	1.09 ± 0.09^c^	miR-29 family protects the kidney from fibrotic damage and linagliptin has been shown to inhibit TGFβ induced epithelial to mesenchymal transition by restoring miRNA-29s level in mouse kidney.	24574044
miR-29c	1 ± 0.08	0.66 ± 0.09^a^	1.01 ± 0.09^b^	0.92 ± 0.06^c^	miR-29 family protects the kidney from fibrotic damage and linagliptin has been shown to inhibit TGFβ induced epithelial to mesenchymal transition by restoring miRNA-29s level in mouse kidney. miR-29c in urinary exosome correlates with both renal function and degree of histological fibrosis.	23946286 24574044
miR-144	1 ± 0.29	0.30 ± 0.13^a^	1.42 ± 0.51	0.58 ± 0.23	miR-144/451 locus previously implicated in erythroid development.	26573221
miR-32	1 ± 0.08	0.65 ± 0.15^a^	1.10 ± 0.14^b^	1.18 ± 0.13^c^	Acts as tumor suppressor in different cancer types.	27323022
miR-451	1 ± 0.34	0.25 ± 0.08^a^	0.91 ± 0.15	0.51 ± 0.22	miR-144/451 locus previously implicated in erythroid development.	26573221
miR-190	1 ± 0.08	0.66 ± 0.13^a^	0.81 ± 0.08	0.64 ± 0.03	Downregulated in renal biopsies obtained from progressive CKD patients.	26707063

Values (fold changes) are given as mean ± SEM; ^a^P < 0.05 5/6 Nx + placebo versus Sham + placebo; ^b^P < 0.05 5/6 Nx + telmisartan versus 5/6 Nx + placebo; ^c^P < 0.05 5/6 Nx + linagliptin versus 5/6 Nx + placebo.

### Integrated miRNA-mRNA analysis

**Figure 3:**
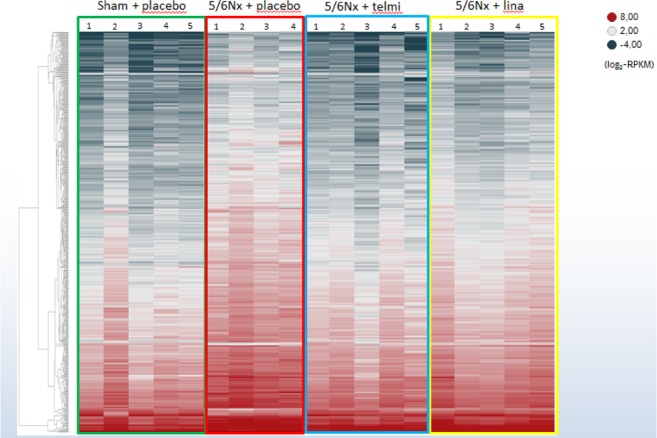
Renal mRNA expression. Heatmap of renal gene expression of sham-operated (green box), placebo-treated 5/6 Nx (red box), telmisartan-treated (blue box) and linagliptin-treated 5/6 Nx (yellow box). Hierarchical clustering of all significantly deregulated mRNAs is depicted according to their log2-transformed expression levels (red = high; dark grey = low).

To identify target mRNAs of the 20 differentially expressed miRNAs in kidneys from placebo-treated 5/6 Nx rats, RNA was sequenced from the corresponding kidney tissues. The principal component analysis revealed that most of the expression profiles of the sham + placebo rats clustered separately to the 5/6 Nx groups whereas the treated 5/6 Nx groups are spread across the 5/6 Nx groups (Supplementary data, Fig. S3). Deregulated mRNA expression is depicted in the heat map in Fig. 3 and all deregulated genes are summarized in Supplementary Table 2. In total, the expression of 1404 genes is deregulated (1.5-fold; *P* < 0.05) in placebo-treated 5/6 Nx rats compared to the sham-operated control rats: the expression of 1015 genes is up-regulated whereas the expression of 389 genes is down-regulated. The effects of telmisartan and linagliptin on these deregulated genes are shown in Fig. 3 and summarized in Supplementary Table 2.

Integrating miRNAs and their corresponding target mRNAs (both 1.5-fold deregulated at *P* < 0.05), resulted in 113 renal miRNA/target mRNA pairs with a Spearman’s correlation coefficient r_s_ < −0.7. The predicted targets displayed in Table 2 are based on either experimental observations or a high level of prediction. Inverse correlations were observed for the four up-regulated miRNAs miR-532-5p, miR-199a-5p, miR-199a-3p and miR-142-3p and the two down-regulated miRNAs miR-203 and miR-29. The effects of telmisartan and/or linagliptin on these identified miRNA/target mRNA pairs are characterized by the loss of Spearman´s correlation coefficient of r_s_

**Table 2:** Differential regulated miRNAs in 5/6 Nx + placebo/+telmisartan/+linagliptin compared to Sham + placebo rats and their corresponding target mRNAs.

miRNA	5/6 Nx + placebo vs. Sham + placebo	5/6 Nx + telmisartan vs. Sham + placebo	5/6 Nx + linagliptin vs. Sham + placebo	Target mRNA	Target prediction	5/6 Nx + placebo vs. Sham + placebo	r_S_	5/6 Nx + telmisartan vs. Sham + placebo	r_S_	5/6 Nx + linagliptin vs. Sham + placebo	r_S_	Function (www.genecards.org)
miR-532-5p	2.46	1.41	1.96	HSPA9	Moderate	0.584	−0.783	1.348	−0.539	1.258	−0.964	This protein plays a role in cell proliferation, stress response and maintenance of the mitochondria.
				VPS37A	Moderate	0.659	−0.733	1.246	−0.576	1.337	−0.624	Required for the sorting of ubiquitinated transmembrane proteins into internal vesicles of multivesicular bodies.
miR-199a-5p	2.30	1.24	1.62	ALS2	High	0.606	−0.867	1.236	−0.309	1.180	−0.733	The protein functions as a guanine nucleotide exchange factor for the small GTPase RAB5.
				ANKRD13C	High	0.625	−0.900	1.305	−0.309	1.261	−0.806	Acts as a molecular chaperone for G protein-coupled receptors.
				ARHGEF28	Moderate	0.664	−0.767	1.230	−0.406	1.120	−0.552	Functions as a RHOA-specific guanine nucleotide exchange factor regulating signaling pathways downstream of integrins and growth factor receptors.
				ARHGEF5	High	0.651	−0.767	1.206	−0.358	1.032	−0.552	The encoded protein may form a complex with G proteins and stimulate Rho-dependent signals. This protein may be involved in the control of cytoskeletal organization.
				CAPRIN1	High	0.553	−0.800	1.287	−0.394	1.383	−0.830	May regulate the transport and translation of mRNAs of proteins involved in synaptic plasticity in neurons and cell proliferation and migration in multiple cell types.
				EDNRB	Moderate	0.480	−0.750	0.784	−0.188	0.556	−0.455	The protein encoded by this gene is a G protein-coupled receptor which activates a phosphatidylinositol-calcium second messenger system.
				FADS6	High	0.367	−0.700	1.909	0.139	1.370	−0.685	Involved in oleate biosynthesis.
				FZD4	High	0.467	−0.717	1.272	−0.297	1.017	−0.576	Involved in the beta-catenin (CTNNB1) canonical signaling pathway, which leads to the activation of disheveled proteins, inhibition of GSK-3 kinase, nuclear accumulation of beta-catenin (CTNNB1) and activation of Wnt target genes.
				GOSR1	High	0.586	−0.833	1.080	−0.333	0.959	−0.661	Involved in transport from the ER to the Golgi apparatus as well as in intra-Golgi transport. It belongs to a super-family of proteins called t-SNAREs or soluble NSF (N-ethylmaleimide-sensitive factor) attachment protein receptor.
				HSPA9	Moderate	0.584	−0.817	1.348	−0.297	1.258	−0.806	This protein plays a role in cell proliferation, stress response and maintenance of the mitochondria.
				JKAMP	Moderate	0.642	−0.800	1.312	−0.564	1.329	−0.564	Facilitates degradation of misfolded endoplasmic reticulum (ER) luminal proteins through the recruitment of components of the proteasome and endoplasmic reticulum-associated degradation (ERAD) system.
				MAGI2	Moderate	0.338	−0.833	1.379	−0.382	1.451	−0.673	Enhances the ability of PTEN to suppress AKT1 activation. Plays a role in nerve growth factor (NGF)-induced recruitment of RAPGEF2 to late endosomes and neurite outgrowth.
				MCFD2	High	0.616	−0.833	1.308	−0.661	1.335	−0.588	The MCFD2-LMAN1 complex forms a specific cargo receptor for the ER-to-Golgi transport of selected proteins. Plays a role in the secretion of coagulation factors.
				NETO2	Moderate	0.421	−0.767	1.318	−0.139	0.937	−0.600	This gene encodes a predicted transmembrane protein containing two extracellular CUB domains followed by a low-density lipoprotein class A (LDLa) domain.
				NT5DC1	Moderate	0.659	−0.700	1.204	−0.309	1.225	−0.879	Miscellaneous.
				PDE4DIP	Moderate	0.558	−0.700	1.443	−0.285	1.159	−0.515	The protein encoded by this gene serves to anchor phosphodiesterase 4D to the Golgi/centrosome region of the cell.
				PPP1R2	High	0.610	−0.767	1.175	−0.176	1.034	−0.576	The protein encoded by this gene binds to the catalytic subunit of protein phosphatase 1, strongly inhibiting its activity.
				SCNN1G	Moderate	0.555	−0.733	1.466	−0.200	1.384	−0.491	Mediates the electrodiffusion of the luminal sodium (and water, which follows osmotically) through the apical membrane of epithelial cells.
				SDPR	Moderate	0.466	−0.817	1.357	−0.345	1.288	−0.709	Plays an important role in caveolar biogenesis and morphology. Regulates caveolae morphology by inducing membrane curvature within caveolae.
				SLC45A3	High	0.595	−0.767	1.698	0.261	1.349	−0.539	Miscellaneous.
				TBC1D14	High	0.567	−0.783	1.271	−0.152	1.259	−0.515	Negative regulator of starvation-induced autophagosome formation.
				VEGFA	High	0.465	−0.800	1.528	−0.224	1.443	−0.721	Growth factor active in angiogenesis, vasculogenesis and endothelial cell growth. Induces endothelial cell proliferation, promotes cell migration, inhibits apoptosis and induces permeabilization of blood vessels.
miR-199a-3p	2.20	1.06	1.07	ARL15	Moderate	0.403	−0.700	1.344	−0.309	1.433	−0.079	Miscellaneous.
				ATP6V1A	Moderate	0.552	−0.783	1.345	−0.091	1.420	−0.358	This gene encodes a component of vacuolar ATPase (V-ATPase), a multisubunit enzyme that mediates acidification of eukaryotic intracellular organelles.
				CAPRIN1	High	0.553	−0.783	1.287	−0.103	1.383	−0.394	May regulate the transport and translation of mRNAs of proteins involved in synaptic plasticity in neurons and cell proliferation and migration in multiple cell types.
				COBLL1	Moderate	0.494	−0.733	1.543	0.055	1.218	−0.212	Miscellaneous.
				DBT	Moderate	0.501	−0.783	1.474	0.212	1.498	−0.442	The branched-chain alpha-keto dehydrogenase complex catalyzes the overall conversion of alpha-keto acids to acyl-CoA and CO2.
				KCNJ13	Moderate	0.439	−0.833	2.294	0.030	1.940	−0.430	This gene encodes a member of the inwardly rectifying potassium channel family of proteins.
				MAP3K5	Moderate	0.556	−0.750	1.380	0.370	1.326	0.030	Serine/threonine kinase which acts as an essential component of the MAP kinase signal transduction pathway.
				MCFD2	High	0.616	−0.767	1.308	−0.164	1.335	−0.127	This protein forms a complex with LMAN1 (lectin mannose binding protein 1; also known as ERGIC-53) that facilitates the transport of coagulation factors V (FV) and VIII (FVIII) from the endoplasmic reticulum to the Golgi apparatus via an endoplasmic reticulum Golgi intermediate compartment (ERGIC).
				NCOA4	Moderate	0.427	−0.767	1.482	0.164	1.611	−0.200	This gene encodes an androgen receptor coactivator.
				PDE8A	Moderate	0.611	−0.783	1.302	−0.042	1.139	−0.055	This PDE hydrolyzes the second messenger, cAMP, which is a regulator and mediator of a number of cellular responses to extracellular signals.
				PTGR2	High	0.611	−0.783	1.545	0.103	1.541	−0.661	This gene encodes an enzyme involved in the metabolism of prostaglandins. The encoded protein catalyzes the NADPH-dependent conversion of 15-keto-prostaglandin E2 to 15-keto-13,14-dihydro-prostaglandin E2.
				PTPRU	Moderate	0.435	−0.767	1.436	0.273	1.587	−0.248	Tyrosine-protein phosphatase which dephosphorylates CTNNB1. Regulates CTNNB1 function both in cell adhesion and signaling.
				RHOT1	Moderate	0.622	−0.767	1.270	0.200	1.292	−0.527	Mitochondrial GTPase involved in mitochondrial trafficking.
				SDC2	Moderate	0.608	−0.700	1.399	−0.297	1.403	−0.673	The protein encoded by this gene is a transmembrane (type I) heparan sulfate proteoglycan and is a member of the syndecan proteoglycan family.
				SEMA3E	Moderate	0.457	−0.700	1.282	0.103	1.305	0.079	Plays an important role in signaling via the cell surface receptor PLXND1. Mediates reorganization of the actin cytoskeleton, leading to the retraction of cell projections.
				SLC33A1	Moderate	0.615	−0.867	1.395	−0.030	1.248	−0.418	Probable acetyl-CoA transporter necessary for O-acetylation of gangliosides.
miR-142-3p	2.17	1.42	2.10	ACSL1	Moderate	0.540	−0.733	1.634	0.139	1.472	−0.697	Activation of long-chain fatty acids for both synthesis of cellular lipids, and degradation via beta-oxidation.
				ATP2A2	High	0.604	−0.883	1.343	−0.685	1.310	−0.685	This gene encodes one of the SERCA Ca(2 + )-ATPases, which are intracellular pumps located in the sarcoplasmic or endoplasmic reticula of muscle cells.
				KCNJ10	Moderate	0.289	−0.767	1.753	−0.491	1.150	−0.661	This gene encodes a member of the inward rectifier-type potassium channel family, characterized by having a greater tendency to allow potassium to flow into, rather than out of, a cell.
				ME1	Moderate	0.595	−0.867	2.030	−0.467	1.864	−0.479	This gene encodes a cytosolic, NADP-dependent enzyme that generates NADPH for fatty acid biosynthesis.
				PDE8A	High	0.611	−0.700	1.302	−0.455	1.139	−0.564	This PDE hydrolyzes the second messenger, cAMP, which is a regulator and mediator of a number of cellular responses to extracellular signals.
				PICALM	High	0.608	−0.800	1.159	−0.406	1.069	−0.697	This gene encodes a clathrin assembly protein, which recruits clathrin and adaptor protein complex 2 (AP2) to cell membranes at sites of coated-pit formation and clathrin-vesicle assembly.
				SLC33A1	Moderate	0.615	−0.717	1.395	−0.455	1.248	−0.733	Probable acetyl-CoA transporter necessary for O-acetylation of gangliosides.
				SYPL1	High	0.641	−0.817	1.175	−0.491	1.179	−0.782	Miscellaneous.
miR-29	0.63	1.15	1.09	ADAM19	High	3.196	−0.750	0.442	−0.297	0.519	−0.200	Participates in the proteolytic processing of beta-type neuregulin isoforms which are involved in neurogenesis and synaptogenesis, suggesting a regulatory role in glial cell.
				ADAMTS7	High	2.117	−0.883	0.590	−0.491	0.553	−0.406	Metalloprotease that may play a role in the degradation of COMP.
				BAK1	High	1.831	−0.767	0.674	0.164	0.786	−0.394	Plays a role in the mitochondrial apoptosic process.
				BEAN1	High	3.688	−0.867	0.271	−0.382	0.274	−0.309	The protein encoded by this gene is one of several proteins that interact with NEDD4, a member of a family of ubiquitin-protein ligases.
				BMP1	High	1.591	−0.700	0.605	−0.127	0.715	−0.248	Cleaves the C-terminal propeptides of procollagen I, II and III. Induces cartilage and bone formation.
				BTG2	High	2.068	−0.733	0.403	−0.430	0.538	−0.418	Anti-proliferative protein; the function is mediated by association with deadenylase subunits of the CCR4-NOT complex.
				C1QTNF6	High	2.791	−0.950	0.481	−0.394	0.571	−0.479	Miscellaneous.
				CCNF	Moderate	3.446	−0.867	0.350	−0.285	0.688	−0.430	Substrate recognition component of a SCF (SKP1-CUL1-F-box protein) E3 ubiquitin-protein ligase complex which mediates the ubiquitination and subsequent proteasomal degradation of CP110 during G2 phase, thereby acting as an inhibitor of centrosome reduplication.
				CCSAP	High	2.038	−0.783	0.570	−0.200	0.589	−0.188	Plays a role in microtubule (MT) stabilization and this stabilization involves the maintenance of NUMA1 at the spindle poles.
				CD276	Experimentally Observed	3.305	−0.867	0.388	−0.115	0.595	−0.333	The protein encoded by this gene belongs to the immunoglobulin superfamily, and thought to participate in the regulation of T-cell-mediated immune response.
				COL11A1	High	2.150	−0.900	0.399	−0.527	0.685	−0.588	This gene encodes one of the two alpha chains of type XI collagen, a minor fibrillar collagen.
				COL1A1	Experimentally Observed	4.282	−0.700	0.389	0.091	0.501	0.188	This gene encodes the pro-alpha1 chains of type I collagen whose triple helix comprises two alpha1 chains and one alpha2 chain.
				COL1A2	Experimentally Observed	2.212	−0.800	0.449	−0.588	0.546	−0.067	This gene encodes the pro-alpha2 chain of type I collagen whose triple helix comprises two alpha1 chains and one alpha2 chain.
				COL3A1	Experimentally Observed	3.562	−0.783	0.298	−0.624	0.436	−0.224	This gene encodes the pro-alpha1 chains of type III collagen.
				COL4A1	Experimentally Observed	1.902	−0.850	0.675	−0.067	0.874	−0.200	This gene encodes a type IV collagen alpha protein. Type IV collagen proteins are integral components of basement membranes.
				COL6A2	High	2.653	−0.733	0.558	0.091	0.684	−0.067	Collagen VI is a major structural component of microfibrils.
				DTX4	High	2.249	−0.917	0.596	−0.164	0.700	−0.200	Regulator of Notch signaling, a signaling pathway involved in cell-cell communications that regulates a broad spectrum of cell-fate determinations.
				DYNLT1	High	1.771	−0.883	0.695	−0.091	0.833	−0.382	Plays a role in neuronal morphogenesis; the function is independent of cytoplasmic dynein and seems to be coupled to regulation of the actin cytoskeleton by enhancing Rac1 activity.
				FAM131B	High	3.386	−0.767	0.144	−0.406	0.632	0.091	Miscellaneous.
				FBN1	Experimentally Observed	2.345	−0.833	0.373	−0.648	0.477	−0.345	Fibrillin-1 is an extracellular matrix glycoprotein that serves as a structural component of calcium-binding microfibrils.
				FCRLA	Moderate	5.053	−0.717	0.374	−0.503	0.469	−0.127	This gene encodes a protein similar to receptors for the Fc fragment of gamma immunoglobulin (IgG).
				HBEGF	High	2.125	−0.850	0.403	−0.382	0.472	−0.673	Growth factor that mediates its effects via EGFR, ERBB2 and ERBB4.
				IL24	Moderate	48.632	−0.900	0.050	−0.418	0.321	−0.418	This gene encodes a member of the IL10 family of cytokines. It was identified as a gene induced during terminal differentiation in melanoma cells.
				ITGA11	High	6.767	−0.817	0.574	−0.115	0.777	−0.103	Integrin alpha-11/beta-1 is a receptor for collagen.
				KLF4	Experimentally Observed	1.757	−0.817	0.490	−0.636	0.521	−0.552	Involved in the differentiation of epithelial cells and may also function in skeletal and kidney development.
				MAP6	High	2.761	−0.867	0.580	−0.055	0.631	−0.285	Involved in microtubule stabilization in many cell types, including neuronal cells.
				MMP2	High	2.823	−0.783	0.404	−0.636	0.561	−0.297	The protein encoded by this gene is a gelatinase A, type IV collagenase, that contains three fibronectin type II repeats in its catalytic site that allow binding of denatured type IV and V collagen and elastin.
miR-29	0.63	1.15	1.09	MPEG1	Moderate	2.891	−0.733	0.309	−0.539	0.406	−0.576	Miscellaneous.
				PDGFRB	High	1.887	−0.700	0.542	−0.164	0.633	−0.103	The protein encoded by this gene is a cell surface tyrosine kinase receptor for members of the platelet-derived growth factor family.
				PIK3R1	Experimentally Observed	2.131	−0.817	0.366	−0.176	0.579	−0.394	Necessary for the insulin-stimulated increase in glucose uptake and glycogen synthesis in insulin-sensitive tissues. Plays an important role in signaling in response to FGFR1, FGFR2, FGFR3, FGFR4, KITLG/SCF, KIT, PDGFRA and PDGFRB.
				PLSCR3	High	1.696	−0.800	0.730	−0.042	0.741	0.030	May mediate accelerated ATP-independent bidirectional transbilayer migration of phospholipids upon binding calcium ions that results in a loss of phospholipid asymmetry in the plasma membrane.
				PMP22	High	1.793	−0.767	0.470	−0.406	0.499	−0.285	Might be involved in growth regulation, and in myelinization in the peripheral nervous system.
				RAB31	Moderate	1.882	−0.717	0.488	−0.358	0.637	−0.309	Required for the integrity and for normal function of the Golgi apparatus and the trans-Golgi network. Plays a role in insulin-stimulated translocation of GLUT4 to the cell membrane.
				RGS16	Moderate	4.951	−0.733	0.198	−0.309	0.364	−0.236	Regulates G protein-coupled receptor signaling cascades.
				SPARC	Experimentally Observed	2.285	−0.900	0.568	−0.139	0.725	−0.188	The encoded protein is required for the collagen in bone to become calcified but is also involved in extracellular matrix synthesis and promotion of changes to cell shape.
				TGFB2	High	2.894	−0.800	0.347	−0.212	0.497	−0.127	This gene encodes a secreted ligand of the TGF-beta (transforming growth factor-beta) superfamily of proteins. Ligands of this family bind various TGF-beta receptors leading to recruitment and activation of SMAD family transcription factors that regulate gene expression.
				TMEM229B	High	2.470	−0.900	0.459	−0.564	0.599	−0.636	Miscellaneous.
				TNFAIP3	High	2.278	−0.867	0.444	−0.588	0.470	−0.770	The protein encoded by this gene is a zinc finger protein and ubiqitin-editing enzyme, and has been shown to inhibit NF-kappa B activation as well as TNF-mediated apoptosis.
				TNFRSF1A	High	2.353	−0.800	0.578	−0.030	0.714	−0.188	Binding of membrane-bound tumor necrosis factor alpha to the membrane-bound receptor induces receptor trimerization and activation, which plays a role in cell survival, apoptosis, and inflammation.
				VASH1	High	2.119	−0.950	0.385	−0.442	0.461	−0.758	Inhibits migration, proliferation and network formation by endothelial cells as well as angiogenesis.
miR-203	0.62	0.75	0.72	AP1S2	High	2.730	−0.850	0.346	−0.176	0.490	−0.867	Subunit of clathrin-associated adaptor protein complex 1 that plays a role in protein sorting in the late-Golgi/trans-Golgi network (TGN) and/or endosomes.
				BCL11B	High	3.109	−0.783	0.441	0.018	0.446	−0.758	Essential in controlling the responsiveness of hematopoietic stem cells to chemotactic signals by modulating the expression of the receptors CCR7 and CCR9, which direct the movement of progenitor cells from the bone marrow to the thymus.
				BIRC5	High	3.097	−0.800	0.577	−0.576	0.819	−0.818	This gene is a member of the inhibitor of apoptosis (IAP) gene family, which encode negative regulatory proteins that prevent apoptotic cell death.
				CCR1	Moderate	4.032	−0.833	0.215	0.297	0.522	−0.673	Receptor for a C-C type chemokine. Binds to MIP-1-alpha, MIP-1-delta, RANTES, and MCP-3 and, less efficiently, to MIP-1-beta or MCP-1 and subsequently transduces a signal by increasing the intracellular calcium ions level.
				CKAP2	High	3.743	−0.983	0.347	−0.564	0.861	−0.855	This gene encodes a cytoskeleton-associated protein that stabalizes microtubules and plays a role in the regulation of cell division.
				CTSS	High	2.408	−0.783	0.464	−0.212	0.664	−0.818	The protein encoded by this gene, a member of the peptidase C1 family, is a lysosomal cysteine proteinase that may participate in the degradation of antigenic proteins to peptides for presentation on MHC class II molecules.
				DYNLT1	Moderate	1.771	−0.767	0.695	−0.685	0.833	−0.879	Plays a role in neuronal morphogenesis; the function is independent of cytoplasmic dynein and seems to be coupled to regulation of the actin cytoskeleton by enhancing Rac1 activity.
miR-203	0.62	0.75	0.72	EDNRA	High	1.803	−0.800	0.533	−0.152	0.590	−0.539	The endothelinA receptor (ETA receptor) is a member of the endothelin receptor group of G-protein-coupled receptors that also includes ETB. They are located primarily in the vascular smooth muscle where they play a role in vasoconstriction and cell proliferation.
				EVI2B	High	3.594	−0.800	0.355	−0.333	0.607	−0.818	Required for granulocyte differentiation and functionality of hematopoietic progenitor cells through the control of cell cycle progression and survival of hematopoietic progenitor cells.
				FGL2	High	2.302	−0.750	0.373	−0.030	0.563	−0.685	The protein encoded by this gene is a secreted protein that is similar to the beta- and gamma-chains of fibrinogen.
				HBEGF	High	2.125	−0.733	0.403	−0.697	0.472	−0.745	Growth factor that mediates its effects via EGFR, ERBB2 and ERBB4.
				IL24	High	48.632	−0.817	0.050	−0.455	0.321	−0.806	This gene encodes a member of the IL10 family of cytokines. It was identified as a gene induced during terminal differentiation in melanoma cells.
				NFIL3	High	2.512	−0.817	0.472	−0.588	0.673	−0.685	Miscellaneous.
				NRG2	High	3.680	−0.833	0.499	−0.430	0.570	−0.394	Direct ligand for ERBB3 and ERBB4 tyrosine kinase receptors.
				NUMBL	High	3.142	−0.833	0.326	−0.055	0.484	−0.612	Plays a role in the process of neurogenesis.
				PCSK2	High	2.655	−0.867	0.331	−0.370	0.604	−0.406	This gene encodes a member of the subtilisin-like proprotein convertase family, which includes proteases that process protein and peptide precursors trafficking through regulated or constitutive branches of the secretory pathway.
				PHLDA3	High	2.373	−0.750	0.639	−0.152	0.670	−0.527	p53/TP53-regulated repressor of Akt/AKT1 signaling. Represses AKT1 by preventing AKT1-binding to membrane lipids, thereby inhibiting AKT1 translocation to the cellular membrane and activation.
				POU2AF1	Moderate	2.930	−0.783	0.465	−0.176	0.549	−0.515	Transcriptional coactivator that specifically associates with either OCT1 or OCT2.
				PRC1	Moderate	3.440	−0.867	0.332	−0.552	0.790	−0.842	The protein is present at high levels during the S and G2/M phases of mitosis but its levels drop dramatically when the cell exits mitosis and enters the G1 phase.
				PRKCB	High	2.753	−0.883	0.416	−0.358	0.457	−0.636	Calcium-activated, phospholipid- and diacylglycerol (DAG)-dependent serine/threonine-protein kinase involved in various cellular processes such as regulation of the B-cell receptor (BCR) signalosome, oxidative stress-induced apoptosis, androgen receptor-dependent transcription regulation, insulin signaling and endothelial cells proliferation.
				RAB31	High	1.882	−0.717	0.488	−0.236	0.637	−0.758	Required for the integrity and for normal function of the Golgi apparatus and the trans-Golgi network. Plays a role in insulin-stimulated translocation of GLUT4 to the cell membrane.
				RELT	Moderate	3.500	−0.700	0.313	−0.067	0.417	−0.830	Mediates activation of NF-kappa-B. May play a role in T-cell activation.
				SCEL	High	3.231	−0.833	0.215	−0.152	0.293	−0.455	May function in the assembly or regulation of proteins in the cornified envelope.
				TGFB2	High	2.894	−0.750	0.347	−0.127	0.497	−0.576	This gene encodes a secreted ligand of the TGF-beta (transforming growth factor-beta) superfamily of proteins. Ligands of this family bind various TGF-beta receptors leading to recruitment and activation of SMAD family transcription factors that regulate gene expression.
				ZC3H12D	High	2.928	−0.933	0.284	−0.467	0.546	−0.818	Miscellaneous.

*miR-29 represents miR-29a/b/c.

< −0.7.

**Figure 4:**
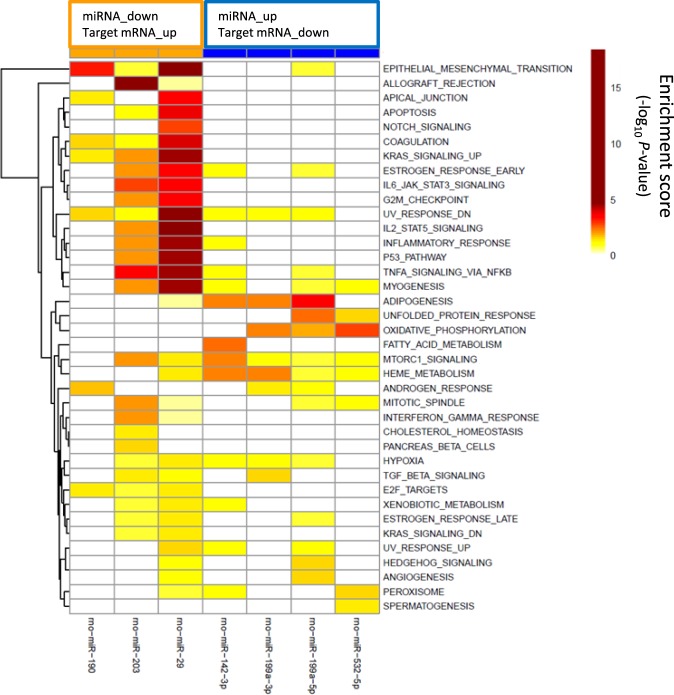
Gene set enrichment analysis of miRNA-mRNA pairs according to 38 hallmark pathways. Effects on distinct pathways are represented by colors indicating enrichment scores. miR-29 represents miR-29a/b/c.

To further characterize the effects of telmisartan and linagliptin on the effects of mRNA-miRNA interactions in 5/6-Nx rats pathway analysis was performed using MSigDB^28^. Hypergeometric testing using the hallmark gene set from MSigDB revealed that the down-regulated miRNAs corresponds to up-regulated target mRNAs that are involved in fibrotic pathways, such as epithelial mesenchymal transition and inflammatory processes, such as TNFα signaling, IL-6/JAK/STAT3 signaling or IL-2/STAT5 signaling (Fig. 4). Down-regulated mRNAs that correspond to up-regulated miRNAs are involved in oxidative phosphorylation and adipogenesis (Fig. 4).

### Urinary exosomal miRNA expression profiling

**Figure 5:**
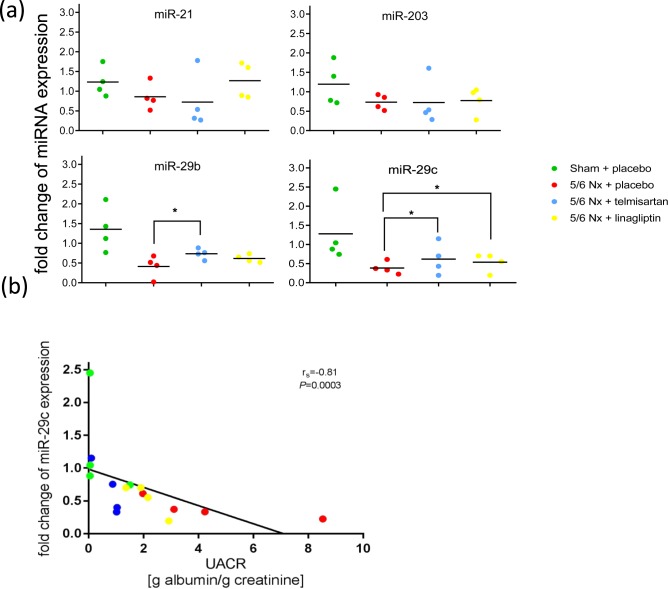
Urinary exosomal miRNA expression. Urinary exosomal levels of miR-21, miR-203, miR-29b and miR-29c in sham-operated (green), placebo-treated 5/6 Nx (red), telmisartan-treated (blue) and linagliptin-treated 5/6 Nx (yellow). (**a**) Relative urinary exosomal miRNA expression analyzed by qRT-PCR was normalized to the mean expression of sham-operated control rats. Significant differences 5/6 Nx + placebo are indicated by *(P < 0.05). (**b**) Correlation between fold change of urinary exosomal miR-29c expression and UACR. Data were compared by Spearman’s correlation coefficient.

To assess which of the aforementioned treatment affected miRNA expression profiles are reflected in urinary exosomes qRT-PCR was employed using TaqMan Array Cards, which contain all deregulated miRNAs described above. Only miRNAs with the highest expression levels in the kidney - miR-21, miR-29b, miR-29c and miR-203 - could be detected in urinary exosomes. The levels of miR-21 and miR-203 were not affected across all groups in urinary exosomes. Urinary exosomal levels of miR-29b and miR-29c were decreased in placebo-treated 5/6 Nx rats compared to the sham-operated group. Telmisartan significantly restored the levels of both urinary exosomal miRNAs miR-29b and miR-29c whereas linagliptin restored the level of only miR-29c compared to the 5/6 Nx placebo group (Fig. 5a). Correlation analysis revealed that down-regulated urinary exosomal miR-29c expression was significantly negatively correlated with increasing UACR (r^2^ = −0.81; *P* = 0.0003, Fig. 5b).

## Discussion

This study shows that the DPP-4 inhibitor linagliptin and the ARB telmisartan induced effects on renal miRNA and their corresponding target mRNA expression in non-diabetic 5/6 Nx rats. Integrating both renal miRNA and mRNA expression profiles enriched pathways, which are deregulated in 5/6 Nx rats, such as fibrotic pathways including epithelial mesenchymal transition and inflammatory processes, such as TNFα signaling, IL-6/JAK/STAT3 signaling or IL-2/STAT5 signaling. In particular, telmisartan and linagliptin exert their strongest effects on fibrotic processes.

Recently it was demonstrated that linagliptin was comparable to telmisartan in preventing CKD progression in non-diabetic, non-glucose dependent 5/6 Nx rats^23^. Tsuprykov *et al*.^23^ demonstrated that linagliptin’s action was pronounced with respect to reduction of renal interstitial fibrosis and glomerular hypertrophy, whereas telmisartan’s beneficial action was mostly due to its blood pressure-lowering effect. In this study, among all 14,605 sequenced mRNAs and 420 detected miRNAs in the kidney both mRNA and miRNA expression levels were not significantly different between telmisartan and linagliptin treated 5/6 Nx rats. Nevertheless, the extent of the beneficial effects exerted by each drug was different. The expression of one gene, namely Renin, was approximately 16-fold (adj- P-value = 0.4) stronger induced in the kidneys of telmisartan treated 5/6 Nx rats compared to linagliptin-treated rats. There was a trend that the beneficial effects characterized by the effects on the deregulated expression levels in 5/6 Nx are more pronounced after telmisartan treatment compared to linagliptin treatment (Table S2). Moreover, a combination of telmisartan and linagliptin might have additive effects as demonstrated by Alter *et al*.^5^. However, this was not investigated in this study and should be considered in future studies. The pharmacological effects of telmisartan and linagliptin are attributed to the interference with multiple pathways. An overlap on gene expression level has been previously described^23^ and confirmed here in addition with miRNA expression level. It is worthwhile to mention that the extent of deregulation is different for individual pathways. Nevertheless, proteomic analyses revealed that post-translational modifications might further differentiate the mechanisms of both drugs^23^. In particular, linagliptin induced a significant upregulation of the HNRNPA1 fragment, an important regulator of the atrial natriuretic peptide-dependent guanylate cyclase pathway, which might contribute to the anti-fibrotic effects. Further investigations are needed to integrate all omics approaches (miRNomics, transcriptomics, proteomics) to further dissect the mode of action of both drugs. In contrast to animal models of diabetic CKD^9,29^ and kidney cell lines exposed to high glucose concentrations^30^ the TGFβ/SMAD2/3 pathway which is a hallmark of renal fibrosis was not directly modulated by linagliptin in the non-diabetic 5/6 Nx model. Nevertheless, indirect modulation of renal fibrosis can occur via pro- and anti-fibrotic miRNAs and our study shows that the degree of deregulation of the renal miRNA expression correlates with increasing interstitial fibrosis.

The renal expression of the up-regulated pro-fibrotic miRNAs miR-150, miR-199a-3p and miR-199a-5p is significantly attenuated after telmisartan or linagliptin treatment. miR-199a-5p is up-regulated during the fibrogenic response to tissue injury^13^ and is a general marker for fibrogenesis in various animal models such as bleomycin-induced lung fibrosis, CCl4-induced liver fibrosis and unilateral ureteral obstruction model of kidney fibrosis^13^. Our study revealed that most of the protein-encoding genes potentially regulated by miR-199a-5p are involved in cytoskeletal organization and cell proliferation processes such as ARHGEF5, VEGFA or SDPR. SDPR encodes Cavin 2, which is involved in maintaining endothelial morphology and function^31^ and inhibition of Cavin 2 expression might cause a loss of cellular integrity and accelerate endothelial mesenchymal transition.

Using different CKD models it was shown that members of the miR-29 family (miR-29s) protect against renal fibrosis by inhibiting endothelial mesenchymal transition and preventing the deposition of ECM^32,33^. The miR-29 family members share a common seed region sequence and are predicted to target the same genes. However, differential regulation of members of the miR-29 family might be a result of the subcellular distribution, suggesting their functional consequence may not be identical. Our study revealed that the expression of miR-29a is not altered between experimental groups. In previous studies it appeared that the three miR-29s may be regulated by distinct mechanisms. In the renal medulla of the Dahl salt-sensitive (SS) rat and a consomic rat strain derived from it, miR-29a is more abundantly expressed compared to miR-29b and miR-29c and the three miR-29 species respond differently to 3 days of a high-salt diet, which might be a consequence of distinct post-transcriptional processing^16^. In addition, our study does not distinguish between miR-29s-3p and -5p-arms. Nevertheless, it is expected that both arms are affected during fibrotic processes in the kidney^8,34^. Predicted target genes for the miR-29 family members largely overlap^35^ which includes genes encoding proteins that are involved in the synthesis of the ECM, including collagen isoforms, laminins, fibrilins, elastins, matrix metalloproteinases, and integrins^36,37^. It is well known that TGFβ/Smad signalling negatively regulates the expression of miR-29s and that DPP-4 inhibitors are able to normalize these changes^9,15^. The present study shows that linagliptin/telmisartan-induced restoration of renal miR-29s expression was accompanied with a decrease in direct target mRNA expression of ECM genes such as COL1A1, COL1A2, COL3A1, COL4A1, COL11A1, FBN1 and MMP2. A similar relation was observed between linagliptin and telmisartan induced changes in miR-29b/c level and collagen type III protein expression (not significant) (Fig. S4). Moreover, normalized miR-29s levels are associated with decreased expression of TGFB2 and BMP1.

In the non-diabetic 5/6 Nx model, CKD progression is more pronounced in 5/6 Nx rats compared to 5/6 Nx mice. This is characterized by a marked increase in renal interstitial fibrosis and markers for glomerular filtration rate, such as cystatin C, in 5/6 Nx rats compared to 5/6 Nx mice. Furthermore, renal gene expression representing markers of renal fibrosis and inflammation, such as COL1A1, COL1A3, TIMP1 and TGF-β, are significantly increased in 5/6 Nx rats compared to 5/6 Nx mice^23,38^. Overall, it seems that mice tolerate the 5/6 Nx surgery better than rats. 5/6 Nx surgery results in only moderate effects on both mRNA and miRNA expression in mice, which might contribute to the different impact in treatment effects of linagliptin between mice and rats. Moreover, the kidney fibrosis phenotype in mice is largely dependent upon the strain specificity^39^. In addition, Srivastava *et al*.^34^ observed discrepancies regarding miR-29s expression level between diabetic mice (STZ treated CD-1 mice) and less fibrotic diabetic 129 Sv mice.

Recent studies revealed that miR-29s levels in urinary exosomes are significantly decreased in CKD patients^14^ and in patients with lupus nephritis^22^. Our study demonstrates that linagliptin/telmisartan induced restoration of miR-29c levels can be demonstrated in urinary exosomes highlighting the importance of urinary exosomes as potential biomarkers for disease progression and monitoring of treatment effects. Moreover, miR-29c levels in urinary exosomes correlate with renal function (albuminuria). One prospective, randomized, controlled study including urine sampling has been completed recently (the MARLINA-T2D trial; Efficacy, Safety and Modification of Albuminuria in Type 2 Diabetes Subjects with Renal Disease with LINAgliptin) to investigate potential short-term albuminuria-lowering effects of linagliptin^40^. In the MARLINA study, individuals at early stages of diabetic kidney disease were included and it was demonstrated that linagliptin significantly improved glycemic control but did not significantly lower albuminuria; in addition, there was no significant change in eGFR^40^. Furthermore, detection of clinically relevant renal effects of linagliptin may require longer treatment. The long-term effects of linagliptin renal outcomes were assessed in the CARMELINA (NCT01897532) study. Urine samples obtained from this study could be used to further substantiate the current findings. The CARMELINA study demonstrated positive effects of linagliptin on progression of albuminuria category - change from non-albuminuria to micro/macroalbuminuria or change from microalbuminuria to macroalbuminuria (P = 0.003)^41,42^. Our latest study indicated that the renoprotective effects of linagliptin cannot solely be attributed to the GLP-1/GLP-1R pathway, highlighting the importance of other signaling pathways influenced by DPP-4 inhibition such as collagen I homeostasis, HNRNPA1, YB-1, thymosin β4 and TGF-β1^36^.

Our study showed that the linagliptin and telmisartan-induced restorative effects on miR-29c expression were reflected in urinary exosomes, suggesting that miRNA profiling of urinary exosomes might be used as a useful biomarker for addressing effects during CKD progression and treatment with drugs, since urinary miRNAs are easily to access. This might be of particular impact for human studies, because in human studies kidney tissue to analyze treatment effects are usually not available.

## Supplementary Information

Supplementary Information2.

Supplementary Information3.

Supplementary Information4.

Supplementary Information5.

Supplementary Information6.

Supplementary Information7.
