# Supplementary figures and images for: Linagliptin and telmisartan induced effects on renal and urinary exosomal miRNA expression in rats with 5/6 nephrectomy
Source: Sci Rep. 2020 Feb 25;10:3373. doi: 10.1038/s41598-020-60336-4 (PMC7042229; doi:10.1038/s41598-020-60336-4)

## Slide 1
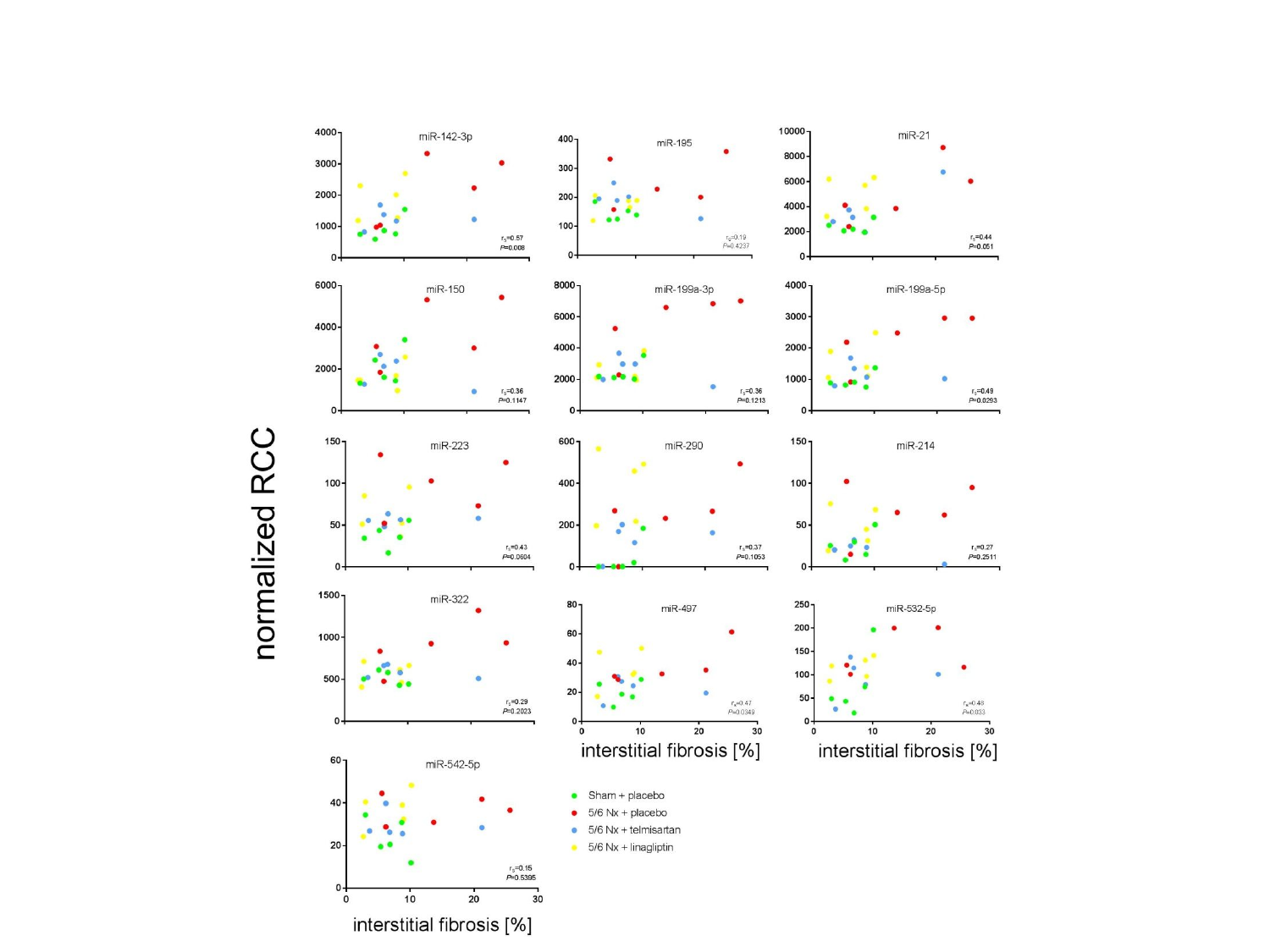

Supplement: Supplementary file 2 — Supplementary Information3. [file 41598_2020_60336_MOESM2_ESM.pptx]

## Slide 1
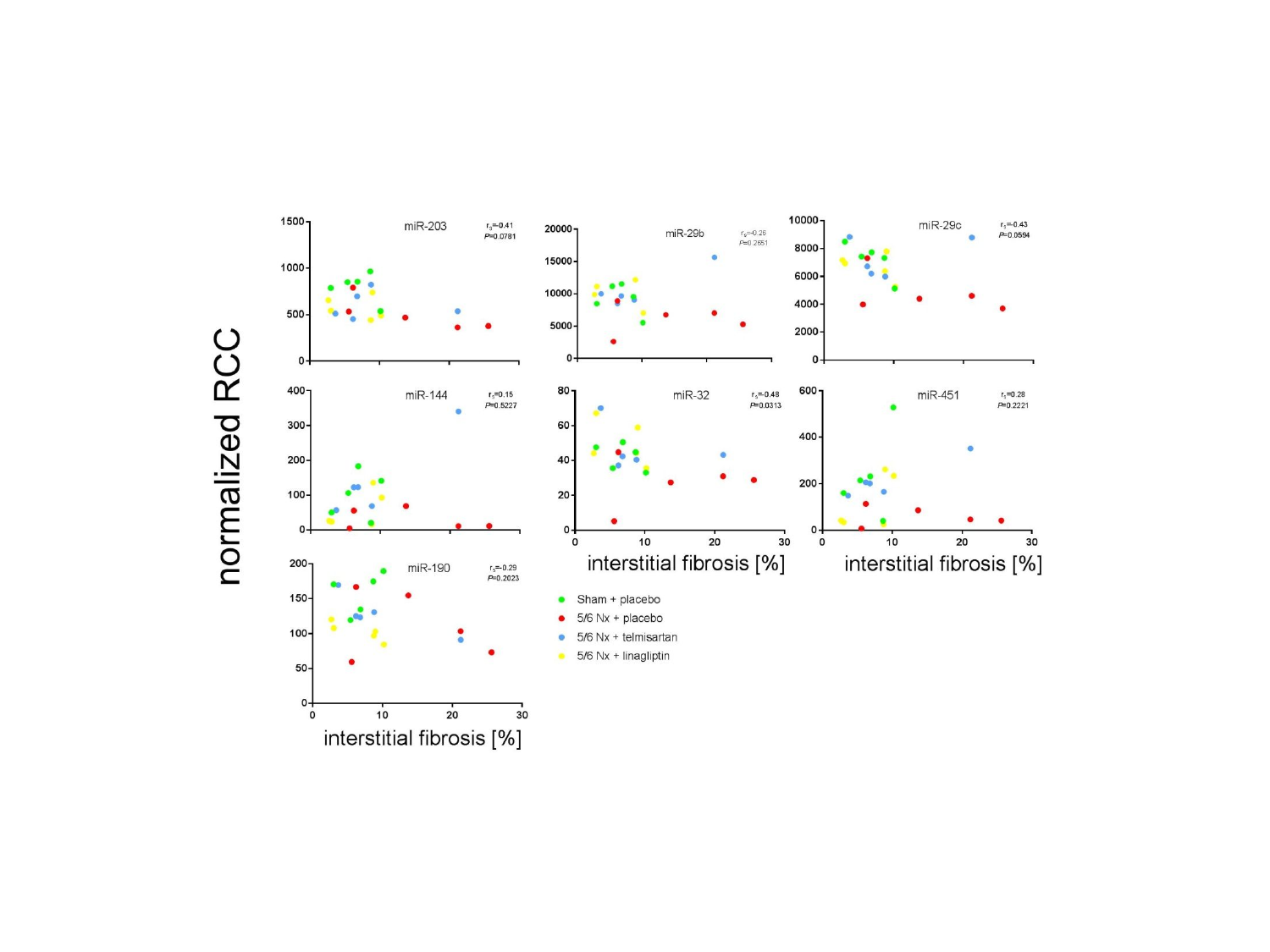

Supplement: Supplementary file 3 — Supplementary Information4. [file 41598_2020_60336_MOESM3_ESM.pptx]

## Slide 1
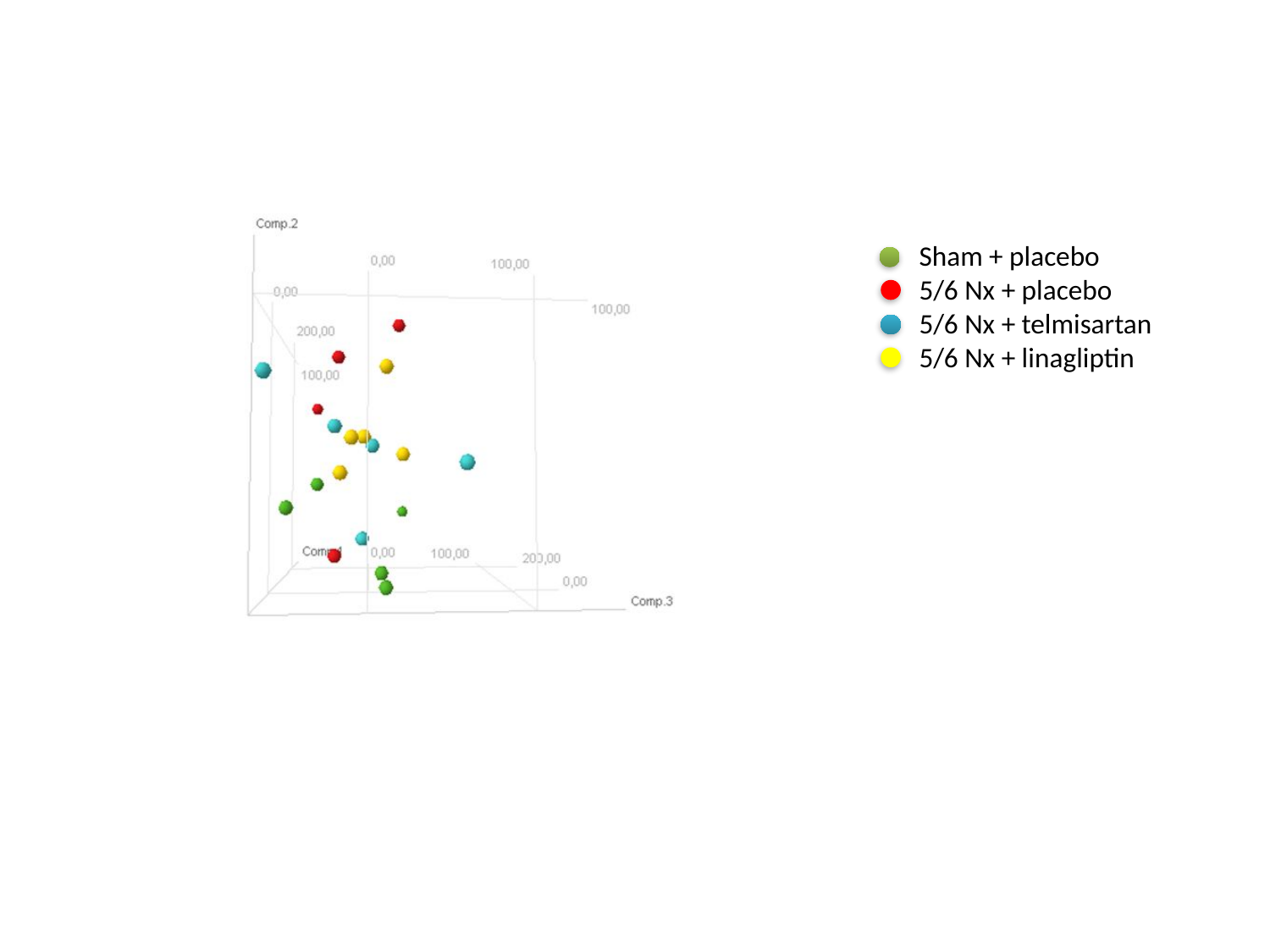

Sham + placebo
5/6 Nx + placebo
5/6 Nx + telmisartan
5/6 Nx + linagliptin

Supplement: Supplementary file 4 — Supplementary Information5. [file 41598_2020_60336_MOESM4_ESM.pptx]

## Slide 1
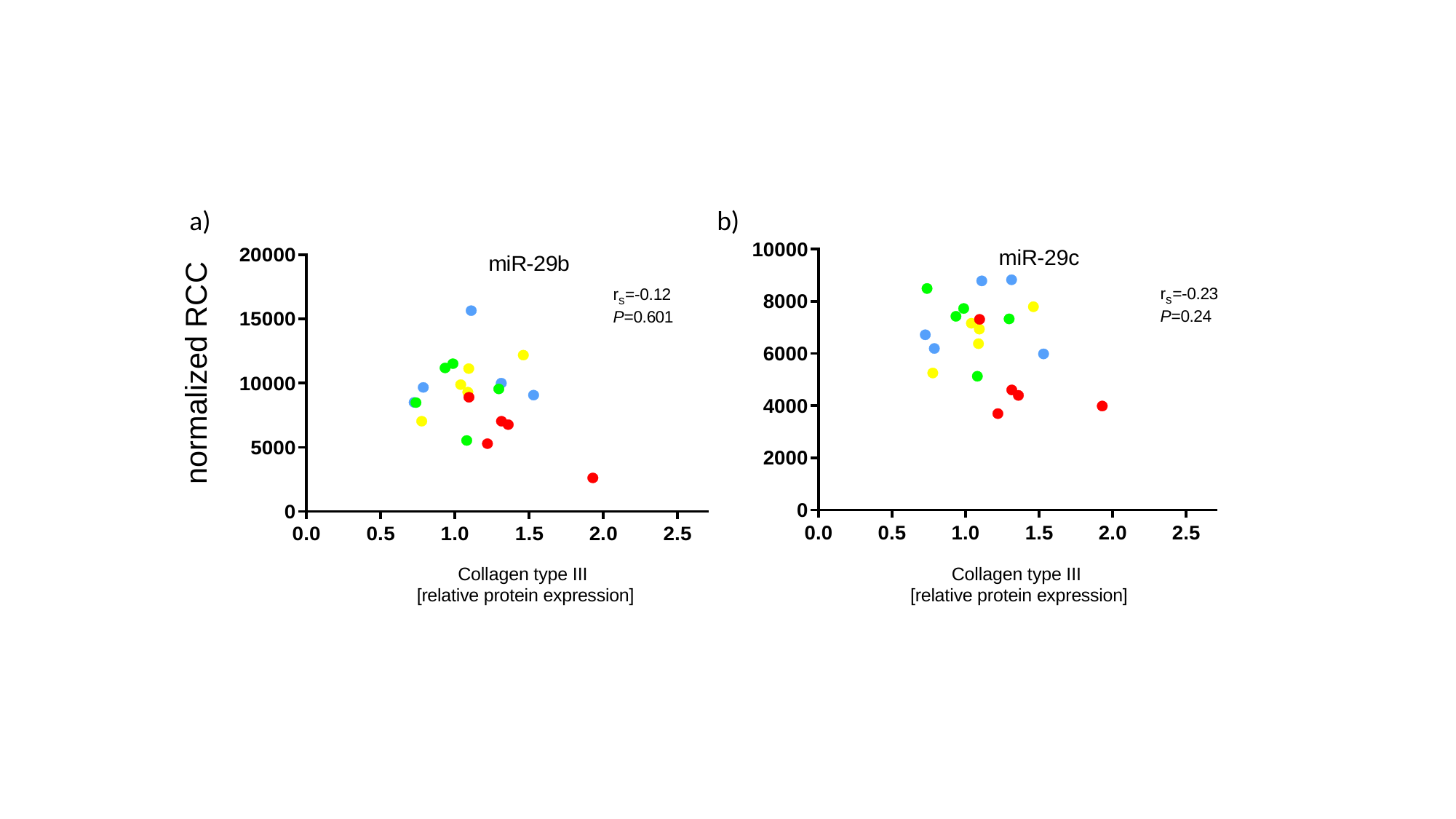

a)
b)

Supplement: Supplementary file 5 — Supplementary Information6. [file 41598_2020_60336_MOESM5_ESM.pptx]
